# Evaluation of Focused Obstetric Ultrasound Examinations by Health Care Personnel in the Democratic Republic of Congo, Guatemala, Kenya, Pakistan, and Zambia

**DOI:** 10.1067/j.cpradiol.2016.11.001

**Published:** 2017

**Authors:** Robert O. Nathan, Jonathan O. Swanson, David L. Swanson, Elizabeth M. McClure, Victor Lokomba Bolamba, Adrien Lokangaka, Irma Sayury Pineda, Lester Figueroa, Walter López-Gomez, Ana Garces, David Muyodi, Fabian Esamai, Nancy Kanaiza, Waseem Mirza, Farnaz Naqvi, Sarah Saleem, Musaku Mwenechanya, Melody Chiwila, Dorothy Hamsumonde, Dennis D. Wallace, Holly Franklin, Robert L. Goldenberg

**Affiliations:** a Department of Radiology, Harborview Medical Center, University of Washington Medical Center, Seattle, WA; b Department of Statistics and Epidemiology, RTI International, Durham, NC; c Department of Obstetrics, Kinshasa University, Kinshasa, Democratic Republic of the Congo; d School of Public Health, University of Kinshasa, Kinshasa, Democratic Republic of the Congo; e Fundación para la Alimentación y Nutrición de Centro América y Panamá (FANCAP), Guatemala City, Guatemala; f Department of Obstetrics and Gynecology, San Carlos University Medical School, Guatemala City, Guatemala; g Fundación para la Alimentación y Nutrición de Centro América y Panamá (FANCAP), Francisco Marroquin University, Guatemala City, Guatemala; h Department of Pediatrics, Moi University School of Medicine, Eldoret, Kenya; i Department of Pediatrics, Moi University School of Medicine, Eldoret, Kenya; j Sonographer, Department of Radiology, Moi University School of Medicine, Eldoret, Kenya; k Department of Radiology, Aga Khan University, Karachi, Pakistan; l Department of Community Health Sciences, Aga Khan University, Karachi, Pakistan; m Department of Pediatrics, University Teaching Hospital, Lusaka, Zambia; n Project Coordinator, Global Network for Women’s and Children’s Health Research, University Teaching Hospital, Lusaka, Zambia; o Lead Sonographer, Radiology Department, University Teaching Hospital, Lusaka, Zambia; p Department of Obstetrics and Gynecology, Columbia University, New York, NY

## Abstract

Prior studies have suggested that obstetrical (OB) ultrasound in low- and middle-income countries has aided in detection of high-risk conditions, which in turn could improve OB management. We are participating in a cluster-randomized clinical trial of OB ultrasound, which is designed to assess the effect of basic OB ultrasound on maternal mortality, fetal mortality, neonatal mortality, and maternal near-miss in 5 low-income countries. We designed a 2-week course in basic OB ultrasound, followed by 12 weeks of oversight, to train health care professionals with no prior ultrasound experience to perform basic OB ultrasound to screen for high-risk pregnancies. All patients with high-risk pregnancies identified by the trainees were referred to higher-level health facilities where fully trained sonographers confirmed the diagnoses before any actions were taken. Although there have been several published studies on basic OB ultrasound training courses for health care workers in low- and middle-income countries, quality control reporting has been limited. The purpose of this study is to report on quality control results of these trainees. Health care workers trained in similar courses could have an adjunctive role in ultrasound screening for high-risk OB conditions where access to care is limited. After completion of the ultrasound course, 41 trainees in 5 countries performed 3801 ultrasound examinations during a 12-week pilot period. Each examination was reviewed by ultrasound trainers for errors in scanning parameters and errors in diagnosis, using predetermined criteria. Of the 32,480 images comprising the 3801 examinations, 94.8% were rated as satisfactory by the reviewers. There was 99.4% concordance between trainee and reviewer ultrasound diagnosis. The results suggest that trained health care workers could play a role in ultrasound screening for high-risk OB conditions.

## Introduction

Prior studies have suggested that the use of obstetrical (OB) ultrasound in low- and middle-income countries (LMIC) could improve OB management by aiding in identification of high-risk conditions.^1^, ^2^, ^3^, ^4^, ^5^ We are participating in a cluster-randomized clinical trial of OB ultrasound under the auspices of the Global Network for Women’s and Children’s Health Research.^6^ The trial is designed to assess the effect of basic OB ultrasound in 5 LMIC on maternal mortality, fetal mortality, neonatal mortality, and maternal near-miss. Eligible pregnant women undergo basic OB ultrasound screening examinations in health center intervention clusters and are referred to participating higher-level facilities if high-risk pregnancies are identified.

Because trained sonographers were not available to staff the rural health centers in which the study ultrasounds were to be performed, the University of Washington’s Department of Radiology (UW) designed a 2-week course in basic OB ultrasound for health care professionals with no prior ultrasound experience.^7^ This course was used to train 41 ultrasound-naïve health care workers (midwives, nurses, radiographers, and medical officers) in 5 LMIC countries to perform basic OB ultrasound to screen for high-risk pregnancies.

The purpose of this article is to evaluate the performance of these 41 trainees. Trained health care workers could have an adjunctive role in ultrasound screening for high-risk OB conditions where access to care is limited. There have been several published studies on basic OB ultrasound training courses for health care workers in LMIC.^1^, ^2^, ^3^, ^4^, ^8^, ^9^, ^10^, ^11^ Although adequate performance after training is crucial,^12^, ^13^ reporting of quality control (QC) activities has been limited. In this study, we report on written and practical testing of 41 trainees and the QC results for 3801 reviewed examinations performed by the trainees during a pretrial training period.

## Methods

The pretrial training period consisted of the following 2 parts: a 2-week intensive course in basic OB ultrasound and a 12-week pilot phase in which trainees performed basic ultrasound examinations on pregnant patients who presented to intervention health centers. The basic OB ultrasound course was administered to the 41 health care workers in the following 5 sites: Karawa, Democratic Republic of Congo; Chimaltenango, Guatemala; Eldoret, Kenya; Karachi, Pakistan; and Lusaka, Zambia. Table 1 displays individual site characteristics. Health care workers were chosen by the study sites to participate as trainees and had no prior ultrasound training. They varied in background, sex, and experience within and between sites (Table 2).

The course was designed to provide the trainees with the knowledge and skills to perform a basic OB ultrasound examination to screen for high-risk pregnancies. The pregnancies considered as high risk were multiple gestation, malpresentation, placenta previa or low-lying placenta, oligohydramnios, polyhydramnios, and cervical insufficiency. When a high-risk pregnancy was identified, trainees were instructed to perform a follow-up ultrasound or to refer the patient to a participating referral facility according to study protocol.

The basic examination consisted of detection of cardiac activity, fetal number, fetal position, amniotic fluid assessment, biparietal diameter (BPD), head circumference (HC), abdominal circumference (AC), femur length (FL), placental position, cervical length at 16-24 weeks gestational age (GA), and detection of some anomalies (ventriculomegaly, anencephaly, hydronephrosis, and spina bifida). Many parameters of the standard second or third trimester ultrasound examination as detailed in the ACR-ACOG-AIUM-SRU Practice Parameter For The Performance of Obstetrical Ultrasound,^14^ such as abnormal heart rhythm and most components of the fetal anatomical survey, were not included in our basic examination.

Approximately one-third of the course time was spent in didactic sessions and two-thirds in supervised hands-on training. The goal was for each trainee to complete 2 supervised ultrasound examinations on each day of the course. These examinations were rudimentary initially, but included all aspects of a basic examination by the fourth day. Trainees, therefore, had hands-on scanning experience with multiple patients by the end of the course. The course was conducted at the study sites by practitioners experienced in ultrasound training. The lead trainer at each site was assisted by local practitioners with substantial ultrasound experience.

During the pilot phase, the trainees performed basic OB ultrasound examinations at intervention health centers. Pregnant women were offered basic OB ultrasound examinations twice during their pregnancies, targeted at 16-22 weeks GA and 32-36 weeks GA. For each ultrasound examination conducted, the trainees saved images on the ultrasound machines depicting fetal position, fetal number, placenta position, cervical length (if 16-24 weeks GA), BPD, HC, AC, FL, and amniotic fluid measurements. The biometry measurements, ultrasound-estimated date of delivery, and final interpretations based on ultrasound findings were summarized on an OB worksheet, and an image of this worksheet was saved for each examination. If fetal or placental abnormalities were discovered by the trainees but were not a part of the routine set of images, images of these abnormalities were also recorded and findings noted by the trainees in a comments section. At least once a week, the images from all the ultrasound machines at each site were downloaded to flash drives and then uploaded to the QC website.

Every ultrasound examination was reviewed and rated on a QC website created for the study by either UW or by local reviewers at each site. Comments were added to individual examinations if indicated. The UW reviewer was a radiologist with 25 years of OB ultrasound experience. The local reviewers were 2 professors of OBs, both of whom conducted OB ultrasound courses at their institutions; a professor of radiology with 10 years of OB ultrasound experience; and 2 lead OB sonographers at their large regional referral hospitals.

**Fig. 1:**
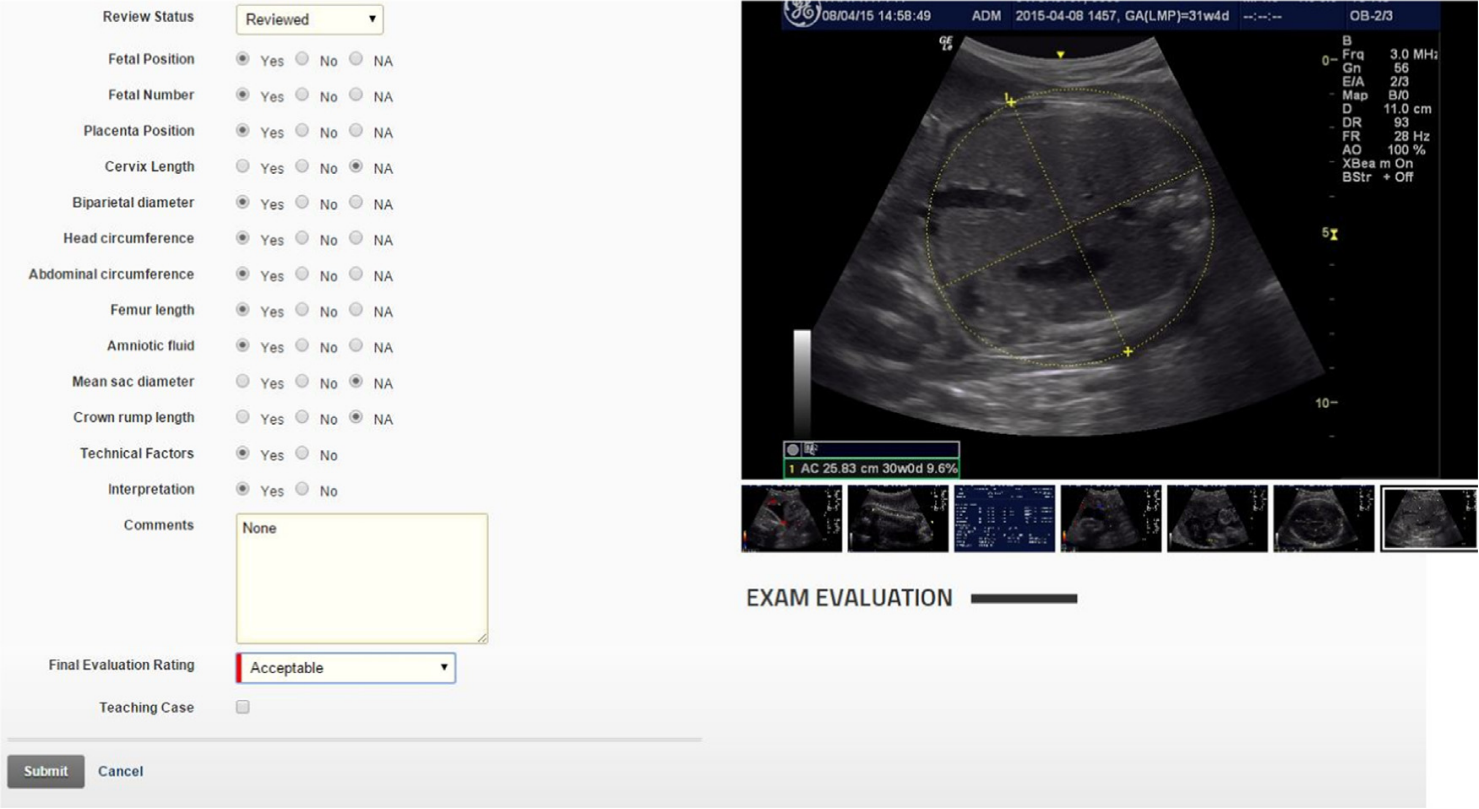
Evaluation web page from QC website. (Color version of figure is available online.)

**Fig. 2:**
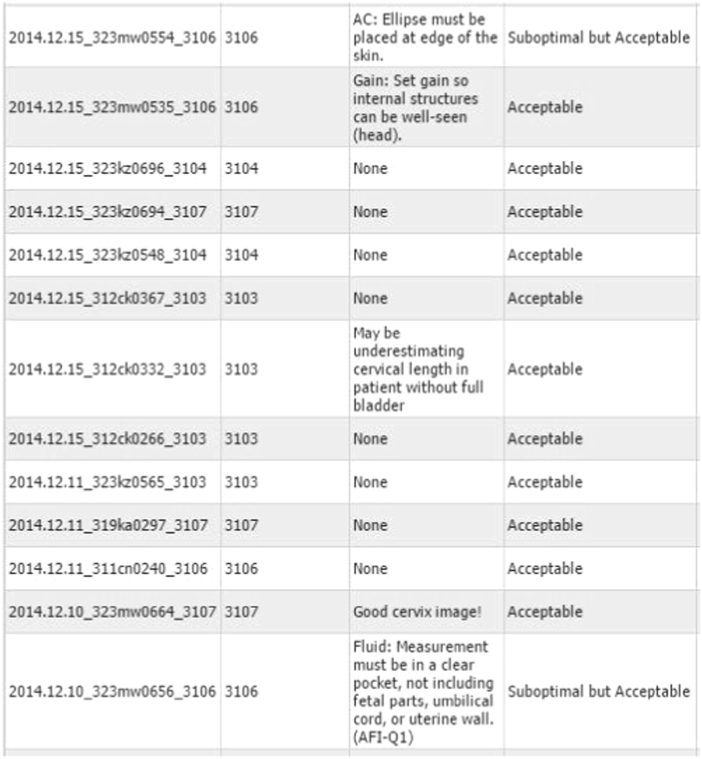
Comments web page from QC website.

Multiple scanning parameters as well as the final interpretations were assessed and scored (Fig 1). All reviewers used the same criteria to rate each component. The reviewers were blinded to the clinical data. After reviews were completed, the examinations with ratings and comments were available for viewing on the website (Fig 2) by the local trainers and trainees. For data analysis, scanning parameter errors are defined as images that do not conform to the criteria established by the investigators for fetal position, fetal number, placental position, cervical length, BPD, HC, AC, FL, amniotic fluid assessment, and technical factors (scanning parameters). Diagnostic errors are defined as errors in interpretation which would have resulted in either inappropriate referral or no referral if referral was indicated.

In addition to feedback from the QC website, the trainees met with the local trainers at least biweekly to discuss their performance. The local trainers observed each trainee scanning at an intervention health center at least biweekly during the pilot period. Conference calls between the UW and the local trainers took place periodically during the pilot phase at each site to discuss the progress of each trainee, using the results of the scanning skills tests, the scan reviews, and direct observation by local trainers. Targeted remedial training was given to some trainees during the pilot period based on the in-person observations, test results, QC results, and conference calls between the local training team and the UW.

Progress during the pilot phase was assessed by QC results as well as by results on practical examinations administered to trainees monthly. In order to obtain certification to participate subsequently in the trial, trainees were required to pass a written examination and a practical examination at the conclusion of the ultrasound course and a practical examination at the conclusion of the pilot phase.

As a part of the QC activities, we conducted replicate secondary reviews for 20 examinations for each of the 5 clinical sites in which both the central reader at the University of Washington and the in-country reviewer in each site replicated those readings, and generated Kappa statistics on the outcome of assessment of acceptability of the intervention. The overall Kappa statistic aggregated across the 5 sites was 0.33 with site-specific values in the 0.3-0.4 range, suggesting that the overall agreement between secondary reviewers could be classified as fair agreement.^15^ No patterns were noted in comparing the central and in-country reviewers, and the percentage of images classified as acceptable by one reviewer and unacceptable by the other being equivalent independent of whether the central reviewer or in-country reviewer found the assessment unacceptable.

## Equipment

The training course and all examinations during the pilot phase were conducted on GE LOGIQ e systems (GE Healthcare, Milwaukee, WI). All patients were scanned transabdominally using wide-band (2.0-5.5 MHz) convex array transducers.

## Patient Population and Approvals

All patients and trainees provided written informed consent to participate. This study was approved by the Ethics Committees of the University of Kinshasa, Kinshasa, Democratic Republic of Congo; the University of Francisco Marroquin, Guatemala City, Guatemala; Moi Teaching and Referral Hospital, Eldoret, Kenya; Aga Khan University, Karachi, Pakistan; and the University of Zambia, Lusaka, Zambia. It was also approved by the Institutional Review Boards of Columbia University, Indiana University, the University of Alabama, Birmingham; the University of Colorado, the University of North Carolina; the University of Washington; and RTI International, Research Park, NC.

## Results

Overall, 97% of the ultrasound examinations were performed between 16 and 36 weeks GA. Because of existing patterns of antenatal care attendance and limited transportation, many examinations took place outside the intended windows of 16-22 weeks and 32-36 weeks (Table 3). High-risk pregnancies were identified by the trainees in 6.7% (255/3801) of examinations. These high-risk pregnancies comprised malpresentation after 32 weeks GA 2.7% (102/3801), multiple pregnancy 1.9% (72/3801), oligohydramnios 0.9% (34/3801), polyhydramnios 0.4% (15/3801), placenta previa after 32 weeks GA 0.3% (11/3801), and others (incompetent cervix, anomalies) 0.5% (19/3801).

### Written and Scanning Skills Tests

Of the 41 trainees who took the written test at the conclusion of the ultrasound course, 39 passed with a score of 75% or greater. The 2 trainees who did not pass on the first attempt did pass on the second attempt 1 week following conclusion of the course.

Of the 41 trainees from all sites who took the scanning skills test at the conclusion of the ultrasound course, 36 passed on the first attempt with a score of 75% or greater. The 5 trainees who did not pass on the first attempt were given additional training by the local trainers and passed the scanning skills test at 1 week or 2 weeks following conclusion of the course. After 12 weeks of the pilot phase, 40 of 41 trainees passed the scanning skills test. The trainee who did not pass the final scanning skills test voluntarily withdrew from the study The mean scanning skills score increased from 78% on the first test to 92% on the fourth test.

### Scan Review

During the 12-week pilot phase, 3801 examinations were evaluated. Further, 73% (2775/3801) were reviewed by UW, and 27% (1026/3801) were reviewed by in-country reviewers. Of the 32,480 images comprising the 3801 examinations, 94.8% were rated as satisfactory by the reviewers (Table 4). There was 99.4% concordance between trainee and reviewer ultrasound diagnosis (Table 5). The 2 most common errors in diagnosis were misdiagnosis of oligohydramnios and placenta previa or low-lying placenta.

## Discussion

Our study reports on quality measures of trainees performing basic OB ultrasound examinations to screen for high-risk pregnancies in rural health centers in 5 LMICs. The trainees underwent a pretrial training period consisting of a 2-week ultrasound course followed by 12 weeks of oversight and rating of all ultrasound examinations they performed. The trainees were rated on multiple scanning parameters for each examination: fetal position, fetal number, placental position, cervical length, BPD, HC, AC, FL, amniotic fluid assessment, and technical factors. The reviewers rated 94.8% of the images obtained by the trainees as satisfactory. There was a 99.4% concordance between the trainees and the reviewers in final ultrasound diagnosis.

Although we were satisfied with the trainees’ performance during the pilot phase, we do not maintain that we trained health care workers with no prior ultrasound experience to be, or function as, sonographers after a 2-week course and 12 weeks of oversight. The trainees performed basic OB ultrasound only to screen for high-risk pregnancies. All patients who screened positive had confirmatory scans from a trained sonographer at a study referral facility. For this type of program to function effectively, it is essential that there be an ongoing strong cooperative relationship between the sites of antenatal care at health centers and higher-level facilities where patients can be appropriately referred.

There have been other published studies on training courses for health care workers in basic OB ultrasound in LMICs, but data on QC activities are limited. A study by Ferraioli and Meloni^8^ to assess the feasibility of an OB ultrasound training course in a developing country setting describes a program in Zanzibar in which 26 radiographers and assistant medical officers were trained in sessions of 6 weeks per year for 5 years . Trainees who completed the course took 3 written and 3 practical tests. In a study by Greenwold et al^1^ to assess the feasibility and sustainability of a basic ultrasound training course in rural Africa, 9 nurses and clinical officers in Mozambique were trained in OB ultrasound in a course with 1 week of lectures and 7 weeks of hands-on training. Of the 1744 examinations performed by trainees, 804 were under supervision and 940 without supervision. The detection rate of most ultrasound variables was similar in the 2 groups. Kimberly et al^2^ trained 21 Zambian midwives in OB ultrasound over 3 training periods of 2-3 weeks to access changes in clinical decision-making. Training was both didactic and supervised hands-on, with unsupervised scanning between training sessions. Twenty-five observed structured clinical examinations were taken by 17 trainees at 2 and 6 months. Of the 441 scans performed during the study period, 214 were reviewed with 96% agreement with fetal heart rate, 91% agreement with placental location, and 70% agreement with BPD. Overall, 2 articles by Rijken et al^9^, ^10^ evaluated the accuracy of fetal biometry performed by locally trained health workers in a refugee camp on the Thai-Burmese border. For biometry between 16 and 40 weeks GA, the health workers, trained in a 3-month course, showed an intraclass correlation of >0.99 in the first study^9^ and low-standard deviation compared with values derived from European and Asian studies in the second study.^10^ Shah et al^3^ trained physicians in Rwanda in a 9-week course covering OBs, cardiac, general abdomen, and procedures, using both lectures and hands-on sessions. In 43% of cases, ultrasound results altered patient management. Of 345 scans, 97 were reviewed, with a concordance rate between trainees and reviewers of 96%. Of scans in all categories, there were 76 true-positive, 18 true-negative, 3 false-positive, and 0 false-negative examinations. Stein et al^4^ report on ultrasound impact in a rural district hospital in Tanzania. Midwives were trained in OB ultrasound in a 2-month course. Of the 148 patients scanned by both trainees and specialist sonographers, there was 100% agreement in identification of twins, fetal heartbeat, and fetal position. In a study by Wylie et al,^11^ 3 mid-level clinical providers in Malawi were given a 1-week ultrasound training course in biometry. Of 178 examinations reviewed, only 5.7% had images considered unsalvageable. There was substantial improvement in the quality of biometry when the first 25% subjects were compared with the last 75%.

Several lessons can be learned from our QC data. Review of placental errors in our study reveal that some trainees had difficulty identifying the lower edge of the placenta relative to the internal os. Oligohydramnios misdiagnosis was secondary to both incorrect measurement and incorrect interpretation of the measurement. Measurement of cervical length is a difficult task for inexperienced ultrasound operators. If this parameter is to be used in the field, didactic and hands-on training must be improved. Adding more hands-on experience as well as modifying didactic material may alleviate some of the scanning challenges the trainees experienced during the pilot phase. Cervical length may be a parameter that some projects may choose not to teach. Although there is disagreement over the worth of transabdominal cervical length measurement, there is evidence that it can be effective as a screening tool.^16^

This study has several limitations. Because the clinical trial is underway, we are blinded to pregnancy outcomes and are therefore unable to report the correlation between the ultrasound findings and outcomes. We hope to publish these data at the conclusion of the clinical trial. The QC evaluations were based only on images submitted by the trainees to the QC website. It is possible that in some instances significant findings were missed on the ultrasound examination and therefore not submitted for evaluation. Concordance between trainee and reviewer diagnosis may be high in part because of the extensive oversight and feedback received by trainees during the pilot. Other programs may not have the resources or infrastructure to undertake this level of oversight.

## Conclusions

Our study demonstrates that after a 2-week course in basic OB ultrasound and a 12-week period of oversight, trainees with no prior ultrasound experience performed basic OB ultrasound examinations independently to screen for high-risk pregnancies and achieved a 99.4% concordance in ultrasound diagnosis with reviewers. The findings suggest that health care workers trained in basic OB ultrasound may be a useful adjunct in ultrasound screening for high-risk OB conditions where access to care is limited. We maintain, though, that patients with identified high-risk pregnancies be referred to higher-level health facilities where fully trained sonographers confirm the diagnoses before any actions are taken.

Feasibility of basic OB ultrasound programs is dependent in part on limiting the cost of training. On-site training is expensive. Exploring the effectiveness of alternative methods such as preservice training^17^ and e-learning^18^ would be of value. It remains to be seen if basic OB ultrasound has an effect in reducing maternal, fetal, and neonatal mortality in LMIC. We hope that the results of clinical trial for which the training program was developed would help answer that question.

## Figures and Tables

**Table 1:** Site characteristics

	Chimaltenango Guatemala	Lusaka Zambia	Western Kenya	Thatta Pakistan	Equateur DRC
Study clusters, *N*	17	10	12	10	6
Annual births 2014, *N*	12,239	6623	7756	10,147	6111

Birth attendant, %					
Physician	42.3	2.5	2.0	22.3	0.5
Nurse/midwife	1.8	55.3	41.9	25.3	68.4
TBA	55.6	25.5	44.1	46.9	27.8
Family/other	0.3	16.7	11.9	5.6	3.3

Birth location, %					
Hospital	39.3	12.4	11.5	27.6	13.9
Clinic	4.5	48.3	31.4	21.9	65.6
Home/other	56.2	39.4	57.1	50.5	20.5

C-section rate, %	18.2	1.1	1.6	7.4	0.2
Neonatal mortality rate (per 1000 live births)	21.1	8.2	13.5	54.7	19.4
Stillbirth rate (per 1000 births)	8.5	21.5	22.8	61.0	16.6
Maternal mortality ratio (per 100,000 live births)	98	119	133	336	403

**Table 2:** Characteristics of ultrasound course trainees

	DRC	Guatemala	Kenya	Pakistan	Zambia
Total, *N*	6	10	12	4	9

Background					
Nurse	6	6	6		
Nurse midwife				4	2
Medical officer		4	6		
Radiographer					7

Sex of trainees					
Male	6	4	4	0	2
Female	0	9	8	4	4
Mean years of professional health care experience (range)	7.3 (4-12)	10.3 (0.8-23)	2.8 (0.7-4)	0.5 (0-1.5)	16.6 (2-39)

**Table 3:** Gestational age at ultrasound during the pilot phase

		Gestational age at ultrasound					
Site	*N*, all ages	<16*N* (%)	16-18*N* (%)	19-22*N* (%)	23-31*N* (%)	32-36*N* (%)	≥37*N* (%)
DRC	997	0 (0.0)	19 (1.9)	74 (7.4)	429 (43.0)	444 (44.5)	31 (3.1)
Zambia	953	1 (0.1)	24 (2.5)	100 (10.5)	514 (53.9)	307 (32.2)	7 (0.7)
Guatemala	914	3 (0.3)	48 (5.2)	95 (10.4)	293 (32.0)	460 (50.2)	17 (1.9)
Pakistan	293	14 (4.8)	24 (8.2)	45 (15.4)	108 (36.9)	101 (34.5)	1 (0.3)
Kenya	664	2 (0.3)	22 (3.3)	55 (8.3)	251 (37.8)	306 (46.1)	28 (4.2)
Total	3821	20 (0.5)	137 (3.6)	369 (9.7)	1595 (41.7)	1618 (42.3)	84 (2.2)

**Table 4:** Scanning parameter errors

Parameter	*N* evaluated	*N* satisfactory	% Satisfactory
Position	3801	3767	99.1
Number	3801	3780	99.4
Placenta	3801	3633	95.6
Cervix	2072	1489	71.8
BPD	3801	3511	92.4
HC	3801	3539	93.1
AC	3801	3585	94.3
FL	3801	3768	99.1
Amniotic fluid	3801	3707	97.5
Total	32,480	30,799	94.8

**Table 5:** Diagnostic errors

Diagnostic errors	False-positive	False-negative	Percentage of diagnostic errors
Abuptio placenta		1	4.8
Fetal presentation	1	1	9.5
Low lying/previa	2	5	33.3
Oligohydramnios	5	4	42.8
Polyhydramnios		1	4.8
Situs inversus		1	4.8
Total	8	13	100
